# Effects of Metal and Metal Oxide Nanoparticles against Biofilm-Forming Bacteria: A Systematic Review

**DOI:** 10.4014/jmb.2403.03029

**Published:** 2024-07-15

**Authors:** Hend Algadi, Mohammed Abdelfatah Alhoot, Anis Rageh Al-Maleki, Neny Purwitasari

**Affiliations:** 1 Postgraduate Center (PGC), Management & Science University (MSU), Shah Alam 40100, Selangor, Malaysia; 2 School of Graduate Studies (SGS), Management & Science University (MSU), Shah Alam 40100, Selangor, Malaysia; 3 Department of Pharmaceutical Sciences, Faculty of Pharmacy, Airlangga University, Surabaya 60115, Indonesia; 4 Department of Medical Microbiology, Faculty of Medicine, University Malaya, 50603, Kuala Lumpur, Malaysia

**Keywords:** Antimicrobial resistance, biofilm, synergistic effects, metal nanoparticles, metal oxide nanoparticles

## Abstract

Biofilm formation by bacteria poses a significant challenge across diverse industries, displaying resilience against conventional antimicrobial agents. Nanoparticles emerge as a promising alternative for addressing biofilm-related issues. This review aims to assess the efficacy of metal and metal oxide nanoparticles in inhibiting or disrupting biofilm formation by various bacterial species. It delineates trends, identifies gaps, and outlines avenues for future research, emphasizing best practices and optimal nanoparticles for biofilm prevention and eradication. Additionally, it underscores the potential of nanoparticles as substitutes for traditional antibiotics in healthcare and combating antibiotic resistance. A systematic literature search, encompassing Web of Science, PubMed, and Google Scholar from 2015 to 2023, yielded 48 publications meeting the review criteria. These studies employed diverse methods to explore the antibacterial activity of nanoparticles against biofilm-forming bacteria strains. The implications of this study are profound, offering prospects for novel antimicrobial agents targeting biofilm-forming bacteria, often resistant to conventional antibiotics. In conclusion, nanoparticles present a promising frontier in countering biofilm-forming bacteria. This review delivers a structured analysis of current research, providing insights into the potential and challenges of nanoparticle utilization against biofilm-related challenges. While nanoparticles exhibit inherent antimicrobial properties with applications spanning healthcare, agriculture, and industries, the review acknowledges limitations such as the narrow scope of tested nanoparticles and the imperative need for extensive research on long-term toxicity and environmental impacts.

## Introduction

In recent years, the pharmaceutical sector has faced a growing imperative to develop treatments targeting biofilms produced by various bacterial species. It is widely recognised that existing traditional methods for bacterial biofilm removal, such as antibiotics, exhibit limited efficacy, with the presence of antibiotic resistance exacerbating this issue [1, 2]. In response to these challenges, researchers have increasingly turned to utilising various forms of nanoparticle-based therapeutic approaches to combat bacterial biofilms, particularly in the field of pharmaceutical science [3, 4]. A biofilm is commonly defined as a cooperative assembly of stationary cells that adhere to each other and the substrate they are affixed to [5-8]. The structure consists of one or more microorganisms encapsulated within a matrix, known as the extracellular polymeric substance (EPS) or extracellular polymeric matrix. The interaction between metal and metal oxide nanoparticles (M/MO-NPs) and biofilm-forming bacteria has garnered significant interest due to its potential to combat microbial resistance and persistent infections. Nanoparticles, particularly those composed of M/MO-NPs, exhibit remarkable antimicrobial properties that offer promising avenues for disrupting biofilm structures formed by various bacterial strains [9, 10].

In recent years, many methods have been developed to introduce nanocomposites consisting of several oxides, significantly enhancing nanoparticles' antibacterial and antibiofilm properties [11, 12]. However, M/MO-NPs have been observed to exhibit significant inhibitory effects on the growth of many types of Gram-positive and Gram-negative bacteria [13, 14]. These options have emerged as promising contenders in addressing the escalating worldwide concern of antibiotic resistance. Nevertheless, comprehensively understanding the mechanism of action of nanoparticles and choosing the most promising nanoparticle materials for future clinical translation persists as challenges due to the inherent variability in nanoparticle manufacturing and testing methodologies [9]. To thoroughly evaluate how biofilm-forming bacteria are affected by M/MO-NPs, an interdisciplinary strategy is essential. Researchers can improve their understanding of the bioactivity and physical properties of the nanoparticles by incorporating knowledge from materials science [15]. Through this multidisciplinary effort, we can learn more about how nanoparticles cause ROS, mechanical damage to cell walls, and metal cation leakage in biofilms. To battle biofilm-related difficulties and antimicrobial resistance, it is vital to understand the physicochemical interactions between nanoparticles and biofilms and the antibacterial capabilities of different M/MO-NPs [9]. Moreover, pharmaceutical sciences play a crucial role in evaluating the therapeutic capacity and compatibility of nanoparticles for use in clinical applications. Nanoparticles, including silver nanoparticles and gold-coated nanoparticles, have demonstrated substantial antibacterial effectiveness against many pathogens [16]. These nanoparticles can be customized to engage with bacterial surfaces, disturb cell membranes, and improve medication delivery [17]. Additionally, researchers have investigated the use of nanomaterials such as mesoporous silica nanoparticles (MSNPs) for drug delivery systems, showing potential in improving the effectiveness of drugs and minimizing their harmful effects [18]. Pharmaceutical sciences aid in comprehending the methods by which drugs work, enhancing the properties of nanoparticles, and ensuring their safety and efficacy for medical applications, contributing to the advancement of novel antimicrobial treatments [19]. Considering environmental impacts allows for the evaluation of ecological consequences and the sustainability of using nanoparticles as antibacterial agents. Such a multidimensional perspective ensures a thorough understanding of the efficacy, safety, and broader implications of employing nanoparticles in combating bacterial biofilms [20].

The rapid development of numerous types of M/MO-NPs is currently underway [4]. It is critical to have a solid grasp of the existing literature on the topic. Furthermore, the efficacy of M/MO-NPs in the fight against bacterial biofilms is discussed by [6, 21, 22]. Currently, the most effective approach is M/MO-NPs, which can suppress biofilm development. However, additional research is necessary to fully understand their potential and address safety concerns. Overall, they show promise in addressing the challenges provided by bacterial biofilms [5, 23].

Systematic Literature Review (SLR) papers are necessary to keep up with the latest updates and identify any research gaps or unresolved issues on a specific topic. A systematic review offers a thorough and contemporary synthesis of existing evidence on the influence of nanoparticles on biofilm development in bacteria. This review facilitates the identification of patterns, deficiencies in knowledge, and domains that require additional investigation.

This review offers several noteworthy contributions. Firstly, it delves into the identification of optimal methodologies and distinct types of nanoparticles with the potential to prevent or eliminate biofilms. Additionally, the comprehensive understanding of the impacts of nanoparticles on biofilms is highlighted, presenting opportunities to advance the development of novel antibacterial agents. Furthermore, the review contributes to the field by creating taxonomies derived from a thorough analysis of the existing literature.

## Materials and Methods

**Fig. 1:**
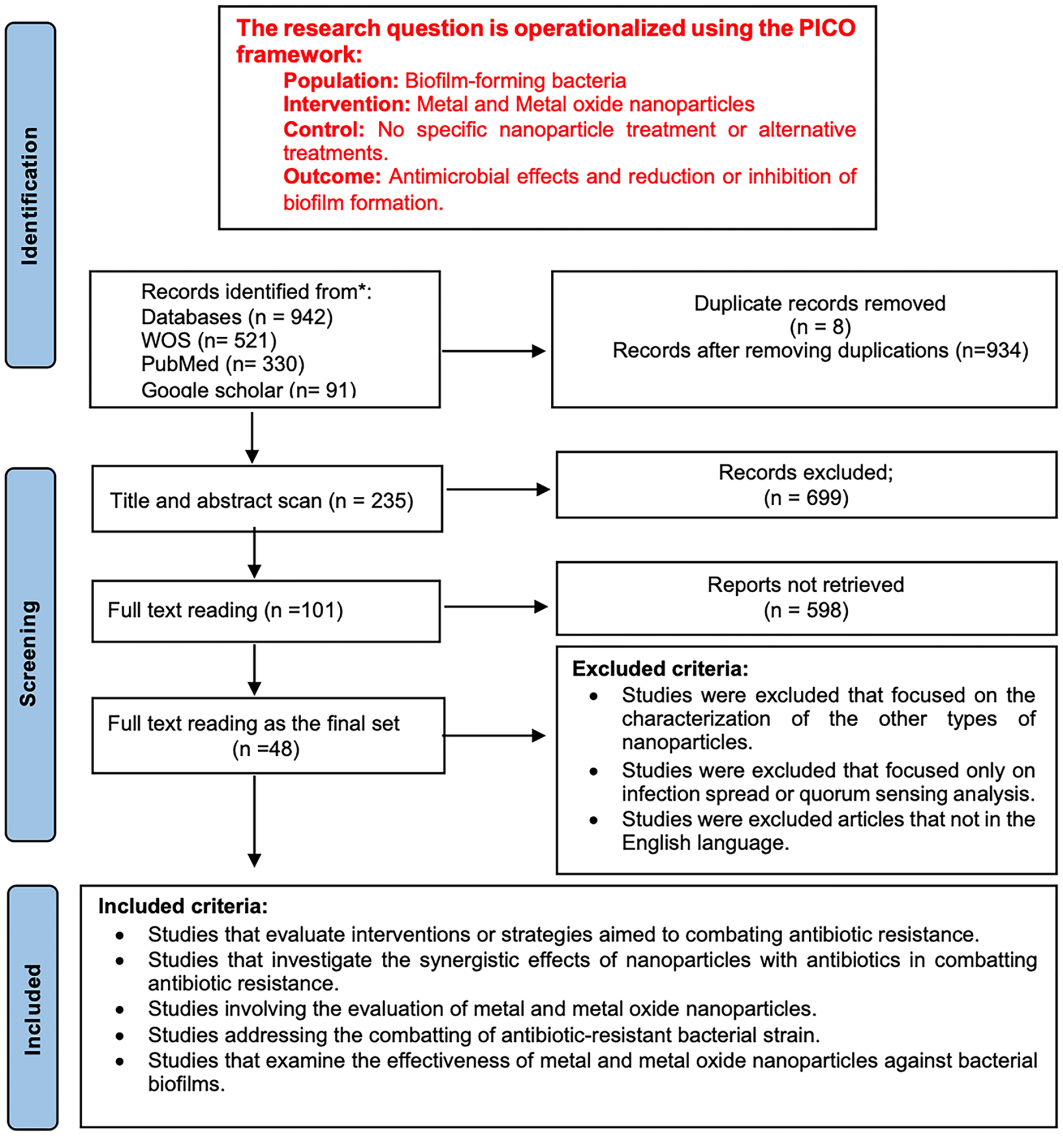
PRISMA flow diagram.

This research aimed to study the effectiveness of M/MO-NPs in inhibiting biofilm formation in bacteria and to identify factors influencing bacterial resistance or susceptibility to these treatments. The SLR utilized diverse and reliable databases rich in high-impact research papers, focusing on keywords such as "Nanoparticles," "Biofilm inhibition," "Bacterial biofilm," "Biofilm disruption," and "Antibiotic resistance" (Fig. 1). The search covered publications from January 2015 to September 2023, retrieved 942 articles from Web of Science, PubMed, and Google Scholar. After filtering duplicates and irrelevant articles, 48 met the inclusion and exclusion criteria. The review process, guided by PRISMA guidelines and detailed in “Fig. 1”, shows the research question operationalized using the PICO framework, sources, screening process, inclusion and exclusion criteria, and the final number of included articles [24].

## Results

### Recent Factors of the Development of Antimicrobial Resistance

**Fig. 2:**
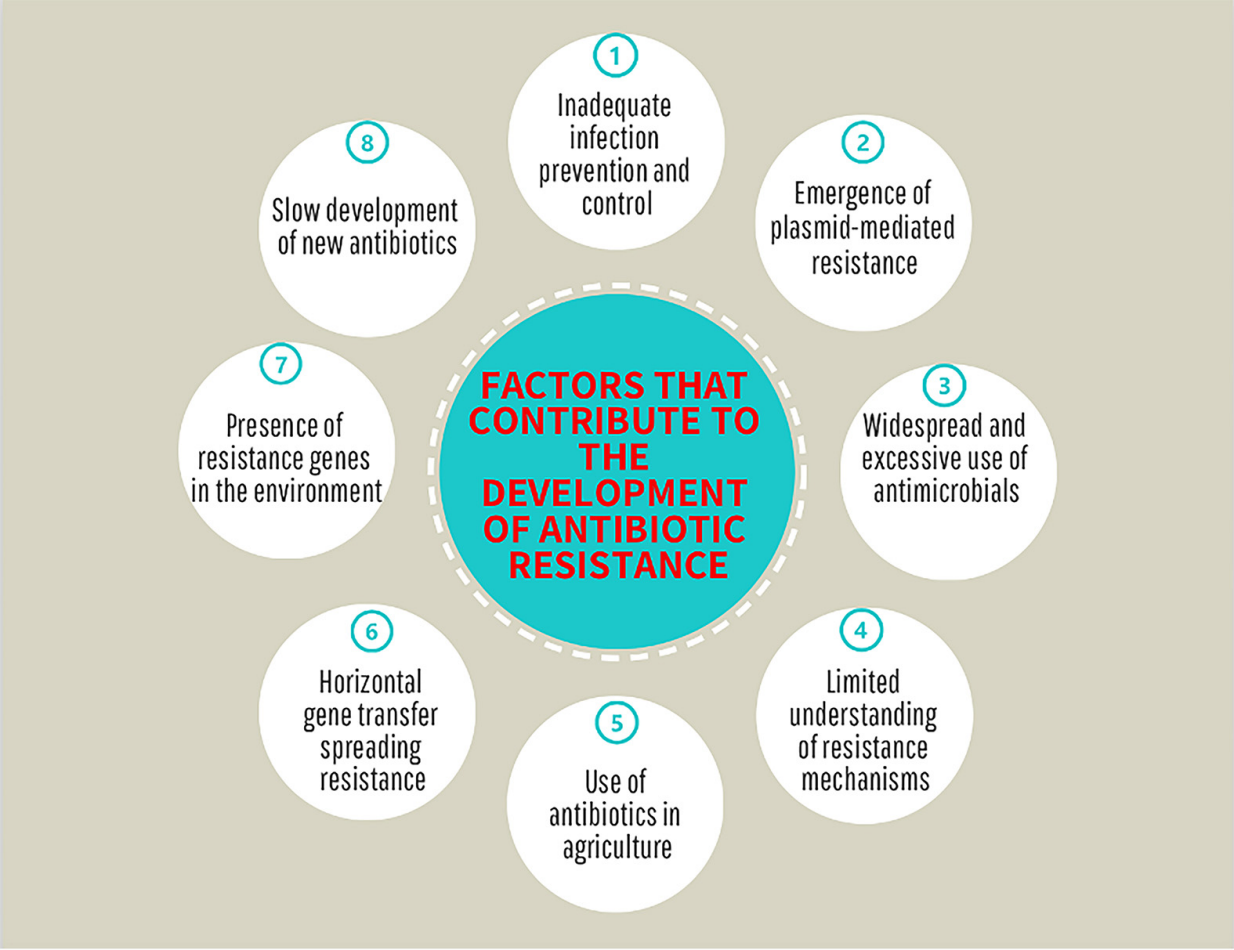
Contemporary factors contributing to the development of antimicrobial resistance.

Several reviews have addressed recent factors contributing to the emergence of antimicrobial resistance (Fig. 2). These factors include the excessive prescription and improper utilisation of antibiotics, widespread application and consumption of antimicrobials, and the presence of potential resistance genes in the environment [25-27]. Additionally, the use of broad-spectrum antibiotics, environmental contamination from the pharmaceutical sector, and implementation of infection control measures in healthcare facilities should be considered [28]. Plasmid-mediated resistance, particularly extended-spectrum β-lactamases (ESBLs), has been notable in antibiotic resistance [29]. Combining nanoparticles with targeting strategies has been suggested to deliver high concentrations of antimicrobial agents to infection sites while minimizing toxicity to non-target cells [30]. The rapid dissemination of resistant bacteria and genes through horizontal gene transfer, coupled with the limited research on antimicrobial resistance across various domains, underscores the importance of understanding these aspects for formulating effective strategies against antibiotic resistance [31].

### Effectiveness of M/MO-NPs in Inhibiting or Disrupting Biofilm Formation

This section highlights recent review articles focusing on the exploration of nanoparticles’ potential in combating biofilm-forming bacteria. Various studies evaluating the effectiveness of nanoparticles in inhibiting the bacterial growth and biofilm formation are summarized in Table 1. These literatures demonstrate the efficacy of M/MO-NPs in inhibiting or disrupting biofilm formation against multidrug-resistant (MDR) pathogens, including *E. coli*, *K. pneumoniae*, *P. aeruginosa*, *A. baumannii*, Methicillin-resistant *Staphylococcus aureus* (MRSA), and *Enterococcus faecalis*, using CuO, Fe_3_O_4_, TiO_2_, ZnO, MgO, Al_2_O_3_ NPs, Ag-NPs, Au-NPs, Si/SiO_2_-NPs and CaO-NPs [3, 6, 14, 15, 17, 25, 27, 32-38]. CuO-NPs generate ROS, damaging bacterial cell walls and membranes, inhibiting biofilm formation. Fe_3_O_4_-NPs penetrate bacterial cells, disrupting biofilm growth through ROS production. TiO_2_-NPs prevent biofilm formation by various bacterial, including *S. aureus* and *P. aeruginosa*, via photocatalytic activity. ZnO-NPs also inhibit biofilm formation by bacteria and fungi. MgO-NPs reduce bacterial adhesion to surfaces, preventing biofilm formation. Al_2_O_3_-NPs are toxic to bacteria, with planktonic cells being more susceptible than biofilms. Ag-NPs and Au-NPs induce oxidative stress through ROS generation. Si/SiO_2_-NPs exhibit antibacterial activity through oxidative stress, bacterial cell damage, and interaction with the bacterial cell membrane. Their high surface area and interaction with bacteria enhance their antibacterial action. Additionally, CaO-NPs exhibit antibacterial activity by generation superoxide on surfaces, interacting with bacterial cell membranes, and potentially leading to bacterial death. However, the effectiveness of these nanoparticles may vary depending on factors such as biofilm maturity, surface composition, nanoparticle size, surface charge, and nanoparticles concentration [15, 17, 34].

### Factors Influencing Bacterial Resistance to M/MO-NPs Treatments and Strategies for Controlling Antimicrobial Biofilms

Several factors influence bacterial resistant or susceptibility to nanoparticle treatments. Bacteria can modify their surface charge to interact with nanoparticles, with Gram-positive bacteria reducing their negative charge through D-alanine integration, while Gram-negative bacteria repel them [8, 32, 36]. Bacterial efflux mechanisms, pigments, and biofilms, which act as physical barriers to nanoparticle efficacy. Nanoparticle shape also affects antimicrobial activity, with triangular shapes causing more damage to bacterial cells than spherical or rod-shaped nanoparticles [18, 38, 39]. However, the antimicrobial activity of nanoparticles is influenced by various factors such as composition, size, surface charge, and shape. In a recent review article, the authors briefly discussed the different control strategies for combating antimicrobial biofilms. These strategies include the use of antibiotics, medicinal plants, nanoparticles, disinfectants, bacteriophages, bioactive glasses, drugs, purified phytochemicals, antimicrobial coatings, physical destruction of biofilm structure, inhibition of EPS production or secretion, degradation of EPS, blocking quorum sensing, and attenuating the production or effectiveness of biofilm virulence factors [10, 15, 16, 33, 40]. Moreover, additional research has identified other techniques, such as using antibodies that specifically target biofilm components and applying physics concepts like nanoparticles and low-intensity electrical current. These tactics aim to prevent the biofilm formation, inhibit bacterial growth, and enhance the effectiveness of antibiotics in treating biofilm infections [29, 41].

### Classification and Diverse Characteristics of Nanoparticles for Enhanced Biofilm Control

**Fig. 3:**
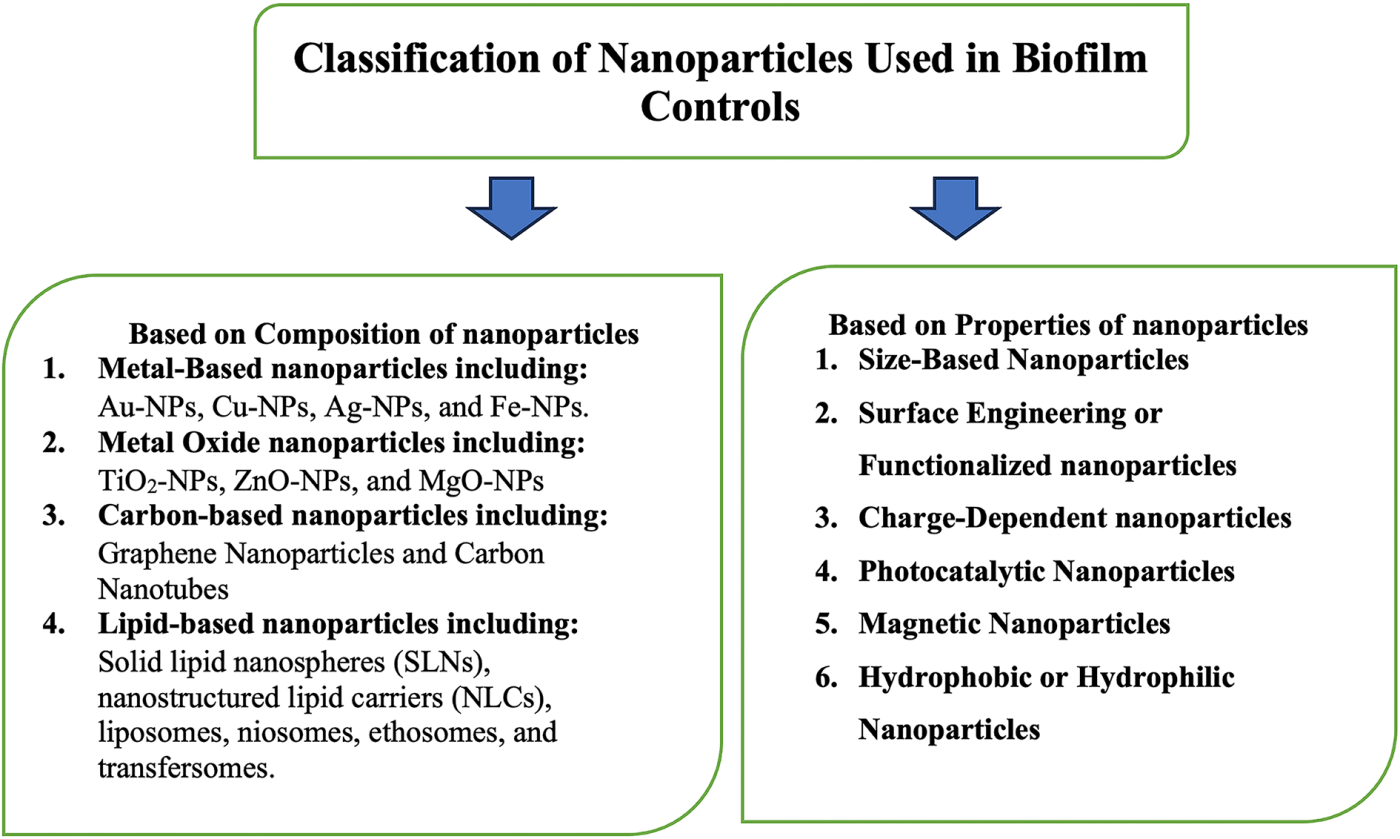
Classification of nanoparticles used in biofilm control.

Nanoparticles used for biofilm control can be categorized based on their chemical makeup and inherent characteristics [8, 21, 38, 42], (Fig. 3). Metal-based nanoparticles (*e.g.*, Au-NPs, Cu-NP, Ag-NP, and Fe-NP), metal oxide nanoparticles (*e.g.*, TiO_2_-NPs, ZnO-NP, and MgO-NP), carbon-based nanoparticles (*e.g.*, graphene nanoparticles and carbon nanotubes), and lipid-based nanoparticles (*e.g.*, solid lipid nanospheres, nanostructured lipid carriers, liposomes, niosomes, ethosomes, and transfersomes) have shown promise in drug delivery systems due to their controlled release capabilities. Nanoparticles can also be categorized based on properties such as size, surface area, morphology, net charge, and physicochemical characteristics. The augmentation of antimicrobial properties has been achieved through the amalgamation of diverse nanoparticles with other substances, leading to the creation of nanocomposites and nanocarriers [43, 44]. The varied characteristics exhibited by nanoparticles make them highly attractive for enhancing the effectiveness of antimicrobial drugs in the managing biofilm infections.

### Synergistic and Enhanced Antibacterial Effects of M/MO-NPs against Resistant Bacterial Infections

In the realm of combating resistant bacterial infections, nanoparticles exhibit synergistic effects and enhanced antibacterial effects when combined with antibiotics. Previous studies suggest that nanoparticles, particularly when paired with aminoglycoside antibiotics, can significantly boost antimicrobial efficacy [37, 45, 46]. For instance, study has demonstrated the synergistic effects of Ag-NPs with aminoglycoside, specifically amikacin, resulting in a notable decrease, 22-fold, in the minimum inhibitory concentration (MIC) of the antibiotic [47]. This combination therapy not only holds promise as a stand-alone antimicrobial treatment but also as an adjunct for combating bacterial infections resistant to multiple drugs. Moreover, nanoparticle-antibiotic combinations, specifically Ag-NPs and ampicillin, have shown potential in reducing the dosage of antibiotics required for treating MRSA infections [47]. This finding suggests the development of novel antimicrobial therapies that are more effective at lower concentrations, thereby minimizing side effects and treatment costs. Nanoparticles have demonstrated superior efficacy compared to traditional antibiotics in treating biofilm-forming bacteria due to their direct interaction with the bacterial cell wall. They disrupt DNA structure, inhibit enzyme function, and deplet ATP production, all of which contribute to their antimicrobial activity [13, 35, 38]. Further studies explore innovative strategies to enhance the antimicrobial activity of nanoparticles. For example, utilizing the synergistic interaction between CuO-NPs and Anthraquinone-2-Carboxylic Acid shows potential in developing combination therapy for *S. aureus* infections [48]. Similarly, functionalizing carbon nanoparticles with oxygen-containing groups and conjugating them with tetracycline has been proposed as a means to construct novel antimicrobial agents with increased antibacterial activity [49].

Additionally, CeO_2_-NPs exhibit robust antibacterial properties against ESKAPE pathogens, notorious for their antibiotic resistance, suggesting their potential as therapeutic agents for infections resistant to conventional antibiotics [50]. ZnO-NPs have also been shown to effectively inhibit the growth of *S. aureus* and reduce the production of enterotoxin A *in vitro*. Furthermore, exposure to ZnO-NPs has been observed to induce morphological alterations and shape distortion in *S. aureus* bacterial cells [46, 51]. This suggests that ZnO-NPs have a significant impact on the structure and integrity of bacterial cells, potentially disrupting their normal morphology and function. These findings underscore the potential of nanoparticles in addressing antibiotic-resistant bacterial infections and offer new avenues for therapeutic intervention.

## Discussion and Future Works

The discussion of the effectiveness of M/MO-NPs against biofilm-forming bacteria has gained significant attention from researchers. Antimicrobial resistance is a complex issue influenced by the overuse and inappropriate prescription of antibiotics. It accelerates resistance development and compromises treatment efficacy, as noted in various studies [15, 25, 27]. Excessive antibiotic use, particularly broad-spectrum antibiotics, contributes significantly to resistance proliferation [28]. Ozdal and Gurkok highlight the significant threat of ESBLs, which can confer resistance to various antibiotics, complicating treatment strategies [18]. Furthermore, Alves-Barroco *et al*, shed light on this phenomenon, emphasizing how this genetic exchange accelerates the dissemination of resistance traits among different bacterial populations [31].

Review articles demonstrate that M/MO-NPs effectively inhibit biofilm formation and exhibit antibacterial activities against MDR pathogens. Various types of M/MO-NPs, including CuO, Fe_3_O_4_, TiO_2_, ZnO, MgO, Al_2_O_3_, Ag-NPs, Au-NPs, Si/SiO_2_-NPs, and CaO-NPs, have shown efficacy across a broad spectrum of pathogens, making them promising agents for combating biofilm-related infections [3, 6, 14, 15, 17, 25, 27, 32-38]. Each type of M/MO-NPs exhibits unique characteristics and mechanisms contributing to hindering or disrupting biofilm growth in diverse ways. Additionally, CuO-NPs and Fe_3_O_4_-NPs exhibit antibacterial effects by generating ROS [36, 52, 53]. While CuO-NPs primarily damage bacterial cell walls and membranes, inhibiting biofilm formation, Fe_3_O_4_-NPs penetrate bacterial cells, disrupting biofilm growth through ROS production [14, 32].

The study by Hochvaldová *et al*., found that TiO_2_-NPs, prevent biofilm formation in *S. aureus* and *P. aeruginosa* through their photocatalytic activity [27]. Moreover, studies have demonstrated conducted that ZnO-NPs and MgO-NPs both exhibit inhibitory effects on biofilm formation. ZnO-NPs demonstrate inhibitory effects on both bacteria and fungi, whereas MgO-NPs reduce bacterial adhesion to surfaces, thus preventing biofilm formation [27, 33, 35]. Additionally, Al_2_O_3_-NP exhibit toxicity towards bacteria, particularly planktonic cells [25]. This research also found that Ag-NPs and Au-NPs induce oxidative stress, generating ROS, thereby exhibiting antimicrobial effects [32]. Furthermore, other studies indicate that Si/SiO_2_-NPs exhibit antibacterial properties through oxidative stress, cell damage, and interaction with bacterial membranes, with their high surface area and interaction enhancing their effectiveness [6, 17, 52].

Furthermore, CaO-NPs have antibacterial properties by producing superoxide and interacting with bacterial cell membranes, potentially causing bacterial death due to their negatively charged surfaces [3]. The efficacy of M/MO-NPs against MDR pathogens has been well-documented, highlighting their broad spectrum of antibacterial properties. This suggests that they have the potential to be useful in tackling the difficulties presented by the creation of persistent biofilms. The aforementioned results underscore the potential efficacy of nanoparticle-based approaches in addressing antibiotic resistance and persistent biofilm-associated illnesses in diverse bacterial strains.

These studies, provide comprehensive insights into the multifaceted nature of bacterial resistance to nanoparticle treatments and the factors influencing the efficacy of these treatments. They highlight the complexity of bacterial resistance to nanoparticles, including the diverse mechanisms used by different bacteria, such as altering their surface charge [8, 32, 36]. This distinction in surface charge alteration showcases the varied responses of different bacterial types, influencing their susceptibility to nanoparticle treatments. Research findings also indicate a reciprocal connection between bacterial cells and nanoparticles, resulting in modifications to the features of both entities and influencing the effectiveness of antibacterial measures. These observations emphasise the complex and interconnected nature of interactions between nanoparticles and bacteria. Furthermore, the findings of these studies suggest that shape-specific interactions between nanoparticles and bacterial cells may offer insights for the design and development of more targeted and efficient antimicrobial strategies [18, 38].

The comprehensive analyses conducted by many studies, offer valuable insights into various approaches employed to address antibacterial biofilms. These strategies comprise a wide range of treatments that aim to target various components of biofilm development, maintenance, and rupture, thereby highlighting the intricate nature of addressing biofilm-related challenges in microbial infections [10, 15, 16, 33]. As discussed by Luo, the study introduces innovative strategies involving physics principles, such as the use of low-intensity electrical currents, and the employment of antibodies specifically designed to target biofilm components. These unconventional approaches highlight the exploration of non-traditional methods to combat biofilm-associated infections [29].

The extensive classification and understanding of nanoparticles for biofilm control provide a foundation for the design and development of tailored antimicrobial agents. This comprehensive approach allows researchers and developers to fine-tune nanoparticle characteristics and combinations to optimize their effectiveness against biofilms, paving the way for innovative and multifaceted strategies in combating biofilm-related infections [8, 21, 38, 42].

Overall, these studies, collectively demonstrate the promising role of nanoparticles, such as Ag-NPs, CuO-NPs, CeO_2_-NPs, and ZnO-NPs, either alone or in combination with antibiotics or other agents, in overcoming antibiotic resistance, disrupting biofilms, and providing new avenues for effective antimicrobial therapy against challenging bacterial infections. These findings highlight the potential of these M/MO-NPs in targeting specific bacterial strains and disrupting their virulence factors [13, 35, 37, 38, 45, 46, 51].

Future studies can leverage the capabilities of M/MO-NPs to address the issue of antimicrobial resistance, hinder the formation of biofilms, and offer innovative approaches for successful antimicrobial treatment against difficult bacterial illnesses. These endeavours exhibit the potential to facilitate the transition of nanoparticle-based methodologies from controlled laboratory environments to real-world clinical settings, hence enhancing patient outcomes and enabling the efficient control of biofilm-associated illnesses.

## Conclusion

The comprehensive investigation into the effects of M/MO-NPs against biofilm-forming bacteria through this systematic review illuminates a promising landscape for combating persistent bacterial infections and addressing antimicrobial resistance. This review contributes to a comprehensive analysis of the efficacy of several types of M/MO-NPs in their ability to inhibit or disrupt the production of biofilms by diverse bacterial species. The review elucidates the optimal strategies and most auspicious nanoparticles for preventing and eradicating biofilms. Furthermore, it highlights the potential of nanoparticles as a viable substitute for conventional antibiotics in healthcare environments and in addressing the issue of antibiotic resistance. The collective evidence gleaned from this systematic review underscores the significant potential of M/MO-NPs whether used alone or in combination with antibiotics or other agents, in overcoming antibiotic resistance, disrupting biofilms, and providing new horizons for effective antimicrobial therapy against challenging bacterial infections. These findings collectively suggest a promising future for applying M/MO-NPs in targeting specific bacterial strains and disrupting virulence factors, indicating a substantial step forward in combatting microbial infections.

## Figures and Tables

**Table 1:** Summary of the mechanisms and advantages of antibacterial activities of Metal/Metal Oxide Nanoparticles (M/MO-NPs).

Classification	Types of NPs	Mechanisms of antibacterial action	Advantages of nanoparticles as antibacterial agents	Target bacteria	Ref
Metal Oxide NPs	CuO-NPs	● ROS generation ● Release copper ions (Cu^2+^) ● physical interactions with bacterial cells	● Effectiveness against antibioticresistant bacteria ● Antioxidant properties for health benefits ● Environmentally friendly synthesis ● Broad-spectrum antibacterial activity	● *MRSA* ● *E. coli* ● Oral bacteria ● *Bacillus subtilis*	[36, 52, 53]
	Fe_3_O_4_-NPs	● ROS Generation by electromagnetic irradiation. ● Penetration and Trojan Horse effect. ● Interaction with biofilm Extracellular Polymeric Substances (EPS). ● Mechanical disruption of biofilm structure.	● Catalytic antibacterial activity ● Moderate antibacterial activity ● Antiplanktonic and antibiofilm activities	● *S. aureus* ● *E. coli* ● *P. aeruginosa* ● *S. epidermidis* ● *Enterococcus hirae* ● *Enterococcus faecalis*	[14, 32, 36]
	TiO_2_-NPs	● ROS generation under UV exposure. ● Interaction with bacterial cell wall. ● Inhibition of bacterial growth.	● Effective antibacterial activity ● Potential against antibiotic resistance ● Effects on bacteria and adaptation ● Photocatalytic properties for enhanced activity	● *S. aureus* ● *P. aeruginosa* ● *E. coli*	[27, 32, 36]
	ZnO-NPs	● Physical damage to bacterial cell membrane. ● ROS production and oxidative stress. ● Inhibition of biofilm formation.	● Antibacterial properties and low toxicity ● Suppression of virulence factors and biofilm formation ● Synergistic effects with antibacterial agents ● Efficiency against human pathogens	● *E. coli* ● *S. pneumoniae* ● *S. aureus*	[32, 33, 35, 36]
	MgO-NPs	● Interaction with spores ● ROS generation and oxidative stress ● Role of defects and vacancies	● Broad bacteriostatic activity ● Effective at low concentrations ● Preferential activity against Gram-Positive bacteria ● Prolonged antibacterial effect	● *E. coli* ● *K. pneumoniae* ● *S. aureus* ● *R. solanacearum*	[27, 33, 36, 52]
	Al_2_O_3_-NPs	● Interaction with bacterial cell wall and membrane ● Penetration and inhibition of cellular processes ● Oxidative breakdown of cell membrane ● Toxicity towards bacteria	● Broad-Spectrum antibacterial efficacy ● Synergistic effects and MDR ● Diverse applications and desirable properties	● *E. coli* ● *P. aeruginosa* ● *P. putida* ● *A. hydrophila*	[25, 35, 36, 52]
	Si/SiO_2_-NPs	● Interactions with Bacterial Cells ● ROS Generation and Disruption of Cell Structures ● Prevention of Biofilm Formation ● Functionalization with Antibiotics	● Minimization of antibiotic side effects and enhanced targeted delivery. ● Specificity in targeting Gram-Positive Bacteria. ● Enhanced delivery to infection sites ● Reduced harmful effects through specific targeting ● Potential benefits against MDR Pathogens	● *S. aureus* ● *E. coli* ● *Acinetobacter baumannii* ● *Citrobacter freundii* ● *Enterobacter* spp ● *E. coli* ● *K. pneumoniae*	[6, 17, 25, 52]
	CaO-NPs	● Superoxide generation ● Induction of oxidative stress and ROS production ● Interaction with bacterial cell membranes ● Changes in cell membrane permeability ● Potential against MDR bacteria	● Enhanced activity with silver doping ● Eco-friendly synthesis methods ● Versatile applications ● Enhanced antibacterial activity from actinomycetes synthesis	● *E. coli* ● *S. aureus* ● *L. monocytogenes* ● *Salmonella typhimurium* ● *Ralstonia solanacearum* ● *Bacillus cereus*	[3, 27, 36, 52]
Metal NPs	Ag-NPs	● Disruption of cellular structures. ● DNA denaturation and inhibition of cellular functions ● ROS production and cellular damage ● Interaction with bacterial cell surfaces	● Antibacterial activity across various bacteria ● High bactericidal efficiency ● Ease of production and modifiability ● Effetive mechanisms of action ● Integration into materials	● *S. epidermidis* ● *E. coli* ● *V. cholerae* ● *S. typhi* ● *P. aeruginosa* ● *S. aureus* ● *B. subtilis* ● *S. pyogenes*	[3, 32, 37, 52]
	Au-NPs	● Interaction with bacterial cell wall and membrane ● ROS generation and oxidative stress ● Interactions with proteins and DNA ● Inhibition of ribosome subunits and ATPase activities.	● Broad-Spectrum antibacterial activity ● Effective against MDR Bacteria ● Enhanced wound healing properties ● Stability and industrial potential	● *B. subtilis* ● *MRSA* ● *E. coli* ● *P. aeruginosa*	[32, 54, 55]
